# Histone chaperone HIRA dictate proliferation vs differentiation of chronic myeloid leukemia cells

**DOI:** 10.1096/fba.2019-00014

**Published:** 2019-08-14

**Authors:** Aditi Majumder, Arya T. Dharan, Ishita Baral, Pallavi Chinnu Varghese, Ananda Mukherjee, Lakshmi Subhadradevi, Geetha Narayanan, Debasree Dutta

**Affiliations:** ^1^ Regenerative Biology Program Rajiv Gandhi Centre for Biotechnology Thiruvananthapuram India; ^2^ Manipal Academy of Higher Education Manipal India; ^3^ Cancer Research Program Rajiv Gandhi Centre for Biotechnology Thiruvananthapuram India; ^4^ Department of Cancer Research Regional Cancer Centre, Medical College Campus Thiruvananthapuram India; ^5^ Department of Medical Oncology Regional Cancer Centre, Medical College Campus Thiruvananthapuram India

**Keywords:** GATA2, hematopoietic precursors, histone variant H3.3, MKL1, proliferation

## Abstract

Abnormal proliferation and disrupted differentiation of hematopoietic progenitors mark leukemia. Histone cell cycle regulator A (HIRA), a histone chaperone, regulates hemogenic to hematopoietic transition involved in normal hematopoiesis. But, its role remains unexplored in leukemia, a case of dysregulated hematopoiesis. Here, the Cancer Cell Line Encyclopedia database analysis showed enhanced *HIRA* mRNA expression in cells of hematopoietic and lymphoid origin with maximal expression in the chronic myeloid leukemia (CML) cell line, K562. This observation was further endorsed by the induced expression of *HIRA* in CML patient samples compared to healthy individuals and Acute Myeloid Leukemia patients. Downregulation of HIRA in K562 cells displayed cell cycle arrest, loss in proliferation, presence of polyploidy with significant increase in CD41^+^ population thereby limiting proliferation but inducing differentiation of leukemia cells to megakaryocyte fate. Induced megakaryocyte differentiation of mouse *Hira*‐knockout hematopoietic progenitors in vivo further confirmed the in vitro findings in leukemia cells. Molecular analysis showed the involvement of MKL1/GATA2/H3.3 axis in dictating differentiation of CML cells to megakaryocytes. Thus, HIRA could be exploited for differentiation induction therapy in CML and in chronic pathological conditions involving low platelet counts.

**Abbreviations**
ALKi: TGF β RI kinase inhibitor VI
AML: acute myeloid leukemia
AML1: acute myeloid leukemia 1
APLF: Aprataxin‐ and PNK‐like factor
ASF1B: anti‐silencing function protein 1 homolog B
BM: bone marrow
BMP4: Bone Morphogenetic Protein 4
BrcAMP: 8‐Bromoadenosine 3′,5′‐cyclic monophosphate
BrdU: 5‐Bromo‐2′‐deoxyuridine
BSA: bovine serum albumin
CABIN1: calcineurin‐binding protein 1
CAF1P60: chromatin assembly factor subunit 1
Ccnd1: Cyclin d1
CD41: Integrin αIIb subunit
CD61: Integrin β3
cDNA: complementary DNA
CML: chronic myeloid leukemia
DAXX: death domain‐associated protein
DMSO: dimethyl sulfoxide
EKLF: erythroid Krüppel‐like factor
ES cells: embryonic stem cells
ETO: eight twenty one oncoprotein
FACS: fluorescence‐activated cell sorting
FACT: facilitates chromatin transcription
FGF2: fibroblast growth factor 2
FLI1: friend leukemia integration 1
G418: geneticin
GATA 2: GATA binding factor 2
GATA1: GATA binding factor 1
GFP: green fluorescence protein
GPIIb: glycoprotein IIb
GPIIIa: glycoprotein IIIa
GSEA: gene set enrichment analysis
GYPA: glycophorin A
H2O2: hydrogen peroxide
HE: hemogenic endothelium
HEK: human embryonic kidney
HIRA: histone cell cycle regulator A
HSC: hematopoietic stem cells
IMDM: Iscove's modified dulbecco's medium
LIF: leukemia inhibitory factor
MKL1: megakaryoblastic leukemia protein‐1
MRTF‐A: myocardin‐related transcription factor‐A
NOD: non‐obese diabetic
PBS: phosphate‐buffered saline
PCNA: proliferating cell nuclear antigen
PI: propidium iodide
PI3K: phosphoinositide 3‐kinase
qRT‐PCR: quantitative real‐time polymerase chain reaction
RIPA: radioimmunoprecipitation assay
RPMI: roswell park memorial institute
RUNX1: runt‐related transcription factor 1
SCID: severe combined immune deficiency
SDS‐PAGE: Sodium dodecyl sulphate‐Polyacrylamide Gel Electrophoresis
TBST: tris buffered saline‐tween 20
UBN1/2: Ubinuclein ½
VEGF: vascular endothelial growth factor

## 1 INTRODUCTION

Leukemia is a disease of the blood or bone marrow (BM). It is characterized by increased numbers of abnormal white blood cells. Specific gene rearrangements or mutations usually result in the formation of new abnormal cellular products, which blocks final differentiation of cells leading to accumulation of immature cells. Leukemia is divided into two types— lymphoid and myeloid.^1^ A very attractive approach to treat myeloid leukemia is called “differentiation induction therapy.” This works as an alternative to killing cancer cells by cytotoxic therapies limiting the exposure to unwanted side effects of cytotoxic chemotherapy. Differentiation therapy is theoretically applicable to all types of leukemia because differentiation block is one of the most important pathophysiology in leukemia.

We reported earlier that histone chaperone histone cell cycle regulator A (HIRA) regulates Runt‐related transcription factor 1 (RUNX1) in hemogenic to hematopoietic transition.^2^ There is also evidence on how HIRA is essential for the β‐*Globin* locus along with other erythropoietic regulators.^3^ Functionally, HIRA acts to incorporate histone variant H3.3 into chromatin in a DNA replication‐independent manner.^4^ The importance of HIRA during mammalian development is evident from the fact that HIRA null mice die between E10 and E11 and display a wide range of phenotypes secondary to defective mesendodermal development.^5^ Other histone chaperones including ASF1B, CAF1P60, and APLF have been implicated in cancer^6^, ^7^, ^8^ but how HIRA could modulate different genes in the context of cancer is poorly understood. Based on our initial observation of HIRA‐mediated regulation of RUNX1 in hematopoiesis, we were intrigued to investigate whether this phenomenon could be relevant in leukemia cells. We found that upon downregulation of HIRA, proliferation of chronic myeloid leukemia (CML) cell line, K562 is significantly reduced while the differentiation potential towards megakaryocyte lineage significantly induced, as demonstrated by in vitro and in vivo analysis. We anticipate that HIRA could be targeted in leukemia cells as a differentiation inducing therapeutic agent.

## 2 MATERIALS AND METHODS

### 2.1 Patient sample

Bone marrow was collected from CML and AML (Acute Myeloid Leukemia) patients (N = 3) from Regional Cancer Centre (RCC), Thiruvananthapuram with their consent following IHEC clearance from both the institutes (RCC: #HEC‐27/2014 and RGCB: #IHEC/01/2015/01). Blood was also collected from normal healthy individuals (N = 4). mRNA was isolated from the whole blood using RNeasy Kit (Qiagen; #74106).

### 2.2 Cell culture

K562 and HL60 cells were cultured in RPMI (Invitrogen; #11875‐093) containing 10% FBS (Invitrogen; 1 600 044), 1% Penicillin/Streptomycin (Invitrogen; #10378016) and 1% Antimycotic/Antibiotic (Invitrogen; #15240062). HEK293T and HCT116 cells were cultured in DMEM supplemented with 10% FBS, 1% Penicillin/Streptomycin, and 1% Antimycotic/Antibiotic.

### 2.3 Quantitative real‐time PCR

Total RNA was extracted using TRIzol reagent (Invitrogen; #15596018) or Qiagen RNeasy Kit according to manufacturer's protocol. cDNA was prepared by a high‐capacity cDNA reverse transcription kit (ABI; #4368814). Power Sybr green master mix (ABI; #4367659) was used for quantitative real‐time PCR (RT‐PCR) analysis. Primer sequences have been enlisted in Table S1.

### 2.4 Western blotting

Cell pellets were lysed in radio immunoprecipitation (RIPA) buffer^2^ and Bradford reagent (Bio‐Rad; 500‐0006) was used to determine the protein concentrations and samples were separated by SDS‐PAGE. Antibodies have been enlisted in Table S2.

### 2.5 RNA interference and generation of stable HIRA‐knocked down cells

Human *HIRA* shRNAs were designed using iRNAi software. Lentiviral vectors containing shRNA targeting human *HIRA* was cloned in the pLKO.1 (Addgene) vector. Lentiviral supernatant was produced in HEK 293T cells by transient transfection using calcium chloride following the protocol described earlier.^2^, ^8^ K562 cells were transfected with viral particles and screened for the generation of stable cells in the presence of puromycin (1 µg/mL) (Sigma; #P8833). After 3 days of transfection, RNA and protein were extracted for analysis. Quantitative RT‐PCR and western blotting confirmed the knockdown of HIRA. We screened two different sets of shRNA‐ #sh and #sh1 where #sh worked best for the knockdown of HIRA in K562 cells.

### 2.6 Cell cycle analysis

1 × 10^6^ K562 cells (control and *HIRA*‐sh) were washed with phosphate‐buffered saline (PBS) and fixed in 70% ethanol. Then cells were treated with RNase A (Sigma; #P4170) and incubated with propidium iodide (PI) (Sigma; #P4170), followed by analysis on BD fluorescence‐activated cell sorting (FACS) AviaTM II instrument.

### 2.7 Ploidy analysis by PI staining and flow cytometry

1 × 10^6^ control and *HIRA‐sh* K562 cells were cytospun on a slide, fixed with 4% paraformaldehyde (Sigma; #P6148) and permeabilized with 0.2% Tween 20 (Sigma; #P1379) in DPBS (Invitrogen; #14190235). Cells were incubated for 15 minutes with 10 µg/mL PI at room temperature. Cover slips were mounted on slides and observed using a confocal microscope. For flow cytometry analysis, around 1 × 10^6^ K562 cells (control and *HIRA*‐sh) were washed with PBS at 500 g for 3 minutes, followed by suspension in ploidy buffer of 4 mmol/L sodium acetate, pH 7.8, RNase A, 0.1% TritonX‐100, 50 µg/mL PI and left to incubate at 37°C for 1 hour. The ploidy in K562 cells was determined by flow cytometry using the instrument mentioned above for cell cycle analysis.

### 2.8 Giemsa staining

Cytospin preparation of 5 × 10^5^ control and *HIRA‐sh* K562 cells were washed with PBS, fixed in ice cold methanol and stained with Wright Giemsa (Sigma; #WG16) solution. Cells were observed and analyzed using light microscopy at different magnification.

### 2.9 Immunofluorescence analysis

Immunostaining to detect expression of HIRA in K562 cells was performed using standard protocols mentioned earlier with minute modification.^2^ The slides were coated with Poly‐L‐Lysine for 1 hour and K562 cells cytospun on the treated slide.

### 2.10 Mouse embryonic stem cell culture

Mouse W9.5 (control) and *Hira* ^−/−^ Embryonic stem cells (ESCs) were cultured in feeder free conditions in ESC medium composed of IMDM (Invitrogen; #12440053), 15% ES qualified serum (Invitrogen; #10439024), 1% antimycotic/antibiotic, 0.0124% mono‐thioglycerol (Sigma; #M6145), 1% penicillin and streptomycin, supplemented with 10^5^ units of LIF/mL (Millipore; #ESG1106) for the maintenance in undifferentiated state.^2^

### 2.11 Cloning and ectopic expression of HIRA cDNA in K562 and HL60 cells

For the ectopic expression of HIRA, *HIRA* cDNA was amplified from K562 cells and cloned within SalI and XhoI sites of pEGFP‐C1 vector (Clontech). Clones were sequenced and used for the transfection of control and *HIRA‐sh* K562 cells. Empty pEGFP‐C1 vector was transfected in control and *HIRA‐sh* cells. Lipofectamine 2000 (Invitrogen; #11668019) was used for transfection and the cells were selected for 14 days in the presence of 250 µg/mL G418 (Sigma; #G8168) and immediately analyzed for the expression of genes investigated in this study.

### 2.12 Ectopic expression of H3.3 in K562

5 × 10^5^ control and *HIRA‐*sh K562 cells were cultured in RPMI containing 10% FBS in 12‐well plate. Cells were transfected with Lipofectamine 2000 and 2 µg of Flag/H3.3‐Flag‐tagged plasmid (Gift from Prof. James J. Bieker) and selected for stable transfection with 250 µg/mL G418 for 14 days.

### 2.13 GFP expression in mouse ES cells

Empty pEGPC1 plasmid was transfected with Lipofectamine 2000 in W9.5 and *Hira* ^−/−^ ES cells and selected with G418 for 15 days for the generation of stable GFP expressing ES cells.

### 2.14 Generation of hemogenic endothelium

GFP^+^ W9.5 (control) and *Hira* ^−/−^ ES cells were cultured in serum and feeder‐free N2B27 medium for 48 hours.^2^ On day 2, 5 ng/mL Bone Morphogenetic Protein 4 (BMP4) (R&D Systems; #314‐BP‐050), 4 ng/mL activin A (R&D Systems; #338‐AC‐010), 12.5 ng/mL Fibroblast Growth Factor (FGF2; R&D Systems; 233‐FB‐025), and 3 µmol/L CHIR99021 (Stemgent; #130‐095‐555) were supplemented in the medium. On day 4, cells were cultured in N2B27 medium supplemented with 20 ng/mL BMP4, 12.5 ng/mL FGF2, 20 ng/mL Vascular Endothelial Growth Factor, 0.25 mmol/L 8‐Bromoadenosine 3',5'‐cyclic monophosphate (BrcAMP) (Sigma; #B5386), and 4 µmol/L SB431542 (TGF‐β RI Kinase Inhibitor VI) (Merck; #616461). At day 6, adherent hemogenic endothelium (HE) cells and floating fraction of hematopoietic precursors were collected.^2^, ^9^

### 2.15 In vivo differentiation of mouse hematopoietic progenitors

Animal work was performed according to the approved protocol #IAEC/636/DSD/2017 and #IAEC/688/DSD/2018 cleared by institutional ethics committee. 1 × 10^6^ control and *Hira* ^−/−^ adherent HE cells and floating hematopoietic precursor cells were suspended in 100 µL PBS and injected in 6 weeks old NOD SCID male mice by tail vein (N = 3). At different time points^10^ drops of peripheral blood (PB) was collected in sterile 2% EDTA/PBS (pH‐8) from tail tip of un‐injected, control and *Hira* ^−/−^ hematopoietic cell injected mice. Mice were imaged regularly by In Vivo Small Animal Imager (IVIS Spectrum, Xenogen). The presence of GFP^+^ cells were analyzed and sorted by FACS in whole blood. 1 × 10^6^ GFP^+^ sorted cells were analyzed for CD41^+^ population and gene expression study was done by qRT‐PCR. At day 31st, mice were euthanized and BM cells from femur bone were collected. GFP^+^ cells from whole BM were sorted and gene expression study was performed by qRT‐PCR.

### 2.16 FACS analysis

Control K562 cells, *HIRA*‐sh K562 cells, control (W9.5) ES cells, *Hira* ^−/−^ ES cells and PB and BM samples isolated from control and *Hira* ^−/−^ hematopoietic progenitor injected mice were analyzed for the presence of CD41 and GFP population by FACS. Cell pellets were washed with FACS buffer [DPBS with 2% (v/v) FBS and 0.263 mmol/L EDTA (Sigma; E9884)]. The cells were suspended in the FACS buffer and incubated with 0.5 µg of fluorescent‐conjugated monoclonal antibody for 1 hour in dark on ice. Following incubation, cells were centrifuged and resuspended in 300 µL of FACS buffer, filtered by cell strainer and subsequently analyzed and sorted by BD FACS AviaTM II. After sorting the cells were collected in basal media with 10% FBS.

### 2.17 BrdU incorporation assay

For the BrdU (Sigma, #B5002) incorporation assay, 2 × 10^5^ vector control and *HIRA*‐sh K562 cells, after day 3 selections with puromycin, were allowed to incorporate 10 µmol/L BrdU for 1 hour in CO2 incubator. Cells were washed twice in FACS buffer at 400 g for 5 minutes. Cells were incubated with 2U DNaseI (Ambion; #AM2222) for 1 hour at 37°C in the dark. Next, cells were fixed with 2% PFA and permeabilized with 0.1% Tween‐20 and washed with FACS buffer. Cells were suspended in primary anti‐BrdU antibody (BD Biosciences #347580) for 1 hour at room temperature followed by incubation with fluorescence‐conjugated secondary antibody anti‐mouse Alexa Fluor 568 (Invitrogen; #A‐11004) for 30 minutes and finally washed and suspended in FACS buffer for further analysis.

### 2.18 Chromatin Immunoprecipitation

Around 10^6^ cells were crosslinked with formaldehyde (1%), and sonicated to generate chromatin fragments. Antibodies were used to immunoprecipitate protein‐DNA cross‐linked fragments. Two microgram of FLAG and GATA2 antibody were used for each sample. Precipitated complexes were eluted and reverse crosslinked. Enrichment of chromatin fragments was measured by qRT‐PCR using Sybr green fluorescence relative to a standard curve of input chromatin. IgG was used as the negative control.^2^, ^8^ List of primers have been enlisted in Table S3.

### 2.19 Statistics

Biological triplicates of the experiments were performed to calculate the statistical significance of the results mentioned in the results section. Student's two‐tailed, unpaired *t* test was used to determine statistical significance. A value of *P* < .05 was considered to be significant. To study the in vivo hematopoietic progenitors differentiation, three biological replicates were analyzed for statistical analysis.

## 3 RESULTS

### 3.1 Increased *HIRA* mRNA expression in leukemia cells

**Figure 1:**
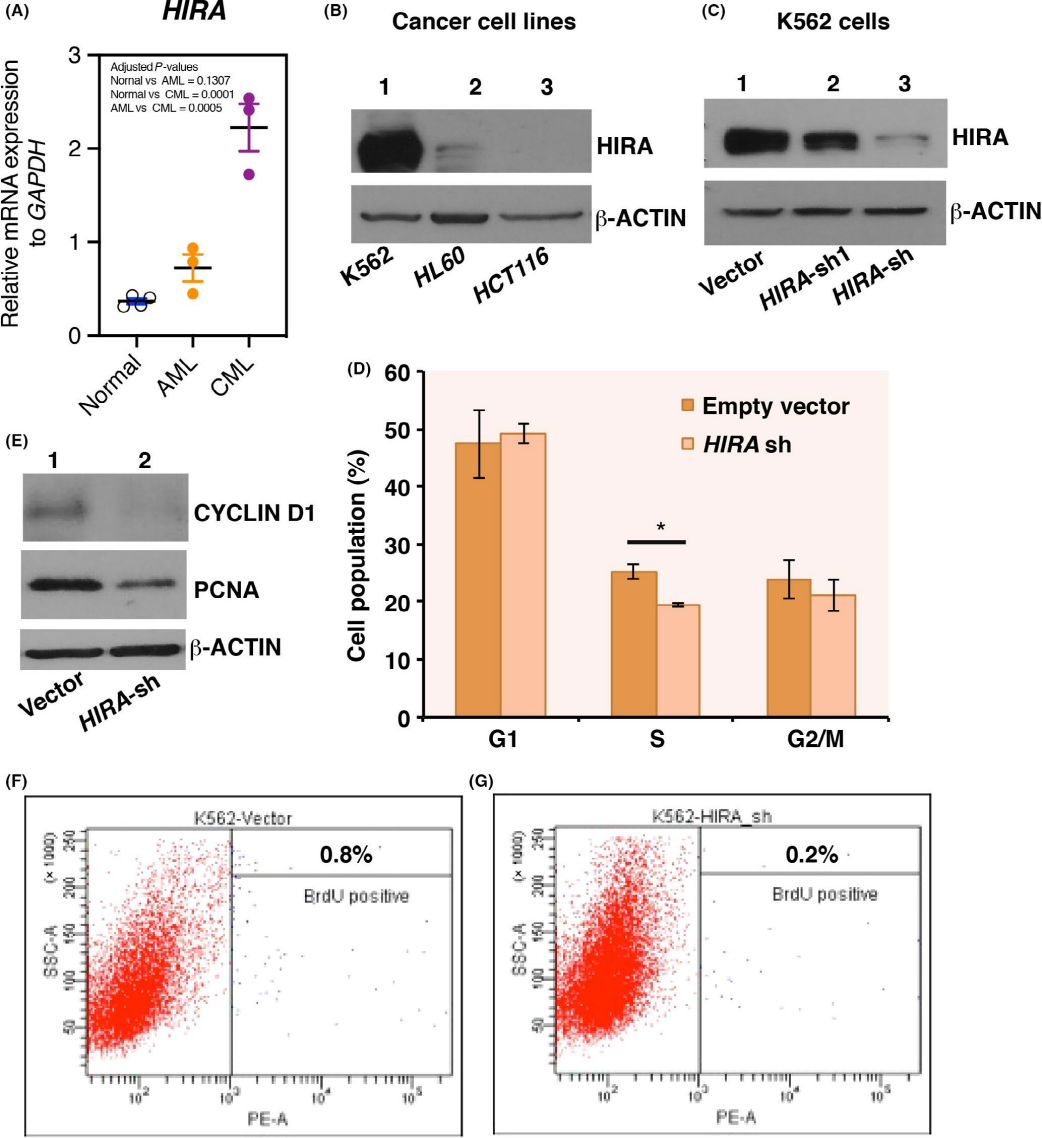
Downregulation of histone cell cycle regulator A (HIRA) inhibit proliferation while induce megakaryocyte differentiation of leukemia cells. (A) Scattered plot represents the expression of *HIRA* mRNA in bone marrow isolated from patients diagnosed with acute myeloid leukemia (AML; N = 3) and chronic myeloid leukemia (CML; N = 3) and in the PB of normal healthy individuals (N = 4). Statistical analysis was performed using one‐way ANOVA with Holm‐Sidak's multiple comparisons test. (B) Western blot analysis for the expression of HIRA in cancer cell lines of CML (K562), AML (HL60) and colon cancer (HCT116) origin. (C) K562 cells were transduced with lentiviral particles expressing shRNA against *HIRA* or empty pLKO.1 vector. Lentiviral vectors containing shRNA targeting human *HIRA* was cloned in the pLKO.1 (Addgene) vector. Two shRNA constructs were used to knockdown HIRA, sh and sh#1. Western blot analysis showed the downregulation of HIRA, wherein #sh construct is more effective. *HIRA*‐sh has been used in the next set of experiments. Vector: transfected with empty pLKO.1 vector and *HIRA*‐sh: *HIRA‐*knockdown K562 cells. (D) Cell cycle analysis was performed after day 3 of puromycin selection and analysis was done on BD Fluorescence‐activated cell sorting (FACS) AviaTM II instrument. The graph represents the percentage of cell population in different phases of cell cycle. Error bar = SEM for three independent experiments. Statistical analyses were performed using Student *t* test function, *P < .05. (E) Western blot analysis for the expression of Cyclin D1 and proliferating cell nuclear antigen (PCNA) in control and HIRA‐sh K562 cells. Experiment was repeated three times and representative blots have been presented. (F, G) Control and *HIRA*‐sh K562 cells were subjected to BrdU incorporation. FACS analysis show the percentage of BrdU‐positive population in control and *HIRA*‐sh K562 cells

Analysis of Cancer Cell Line Encyclopedia (CCLE)^11^ for *HIRA* mRNA expression indicated enhanced mRNA expression in cells derived from hematopoietic and lymphoid lineages (Figure S1). RNA‐seq data analyzed by the CCLE algorithm showed CML cell line, K562, has the maximal expression with a value of 5.273 (Figure S1). K562 cell line represents blast crisis stage with limited differentiation potential.^12^ Next, we compared the expression of HIRA in healthy individuals vs patient samples of CML and AML origin. *HIRA* mRNA expression was significantly enhanced in the CML patients than in normal or AML samples (Figure 1A). Western blot analysis in cell lines of CML (K562) and AML (HL60) origin along with a colon cancer cell line (other than hematopoietic or lymphoid origin) demonstrated enhanced level of HIRA in CML cell line (Figure 1B). So, K562 cells constitute an ideal model to study the role of HIRA in leukemia cells.

### 3.2 Downregulation of HIRA inhibits proliferation and induces differentiation

ShRNA construct, #sh, effectively downregulated HIRA in K562 cells (Figure 1C, Figure S2A). In silico analysis detected no off‐targets for these human *HIRA* shRNAs^13^ (Figure S2B). Cell cycle analysis demonstrated significant reduction in S‐phase population upon downregulation of HIRA in K562 cells (Figure 1D). Cell cycle proliferation marker Cyclin D1 and Proliferating cell nuclear antigen was significantly downregulated in *HIRA‐sh* K562 cells (Figure 1E). Reduced cell population in S‐phase indicates arrest in proliferation. To investigate the possibility of this cellular arrest, we measured the incorporation of 5‐bromo‐2'‐deoxyuridine (BrdU, a synthetic thymidine analogue) by control and *HIRA*‐sh K562 cells. Proliferating cells can incorporate BrdU in S‐phase of the cell cycle.^14^ Cells were pulsed with BrdU, and the level of BrdU incorporation was determined by FACS analysis. BrdU‐positive population reduced from 0.8% in control to 0.2% in *HIRA*‐sh K562 cells. (Figure 1F,G). So, we could demonstrate that downregulation of HIRA indeed inhibited the proliferation of K562 cells.

**Figure 2:**
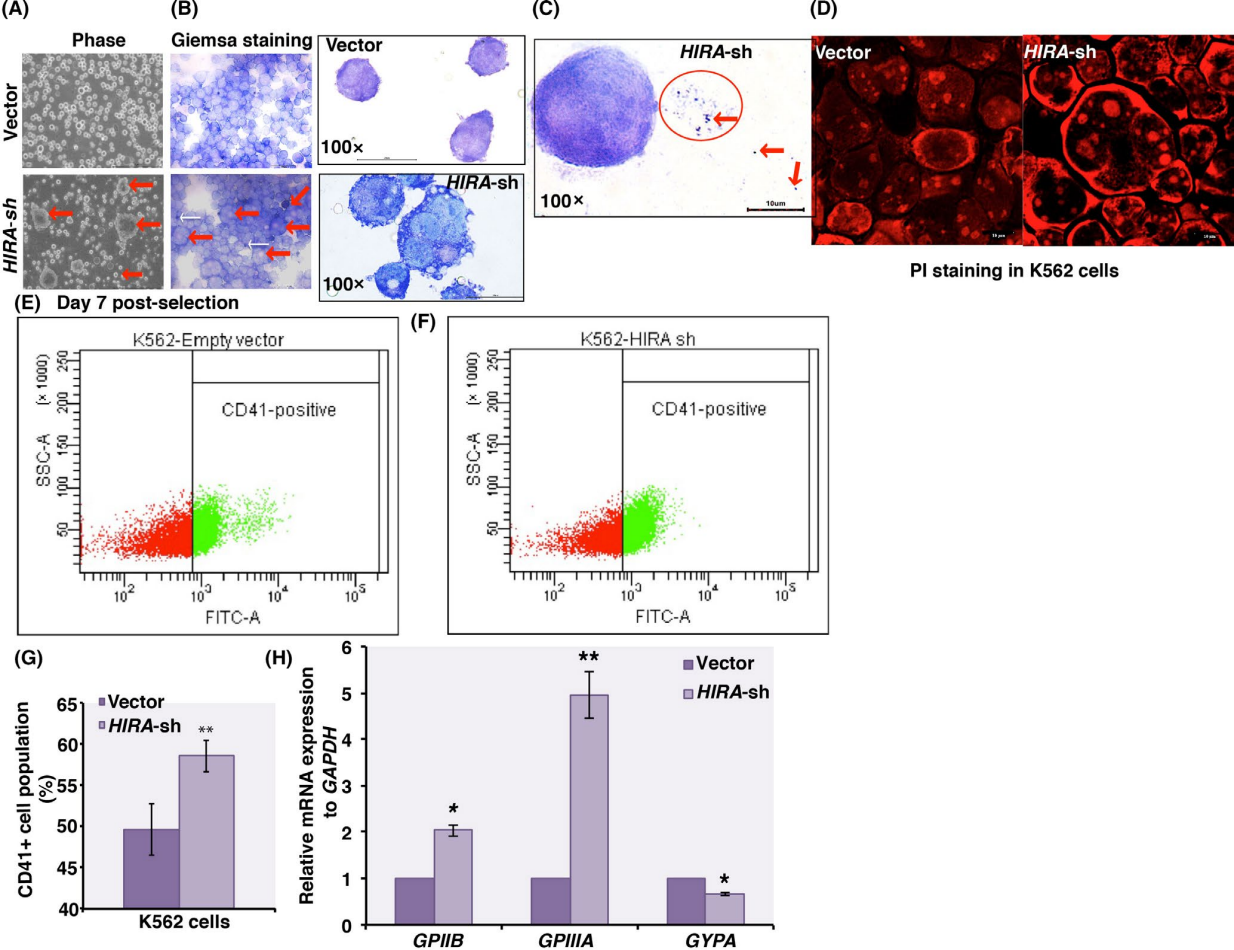
Downregulation of histone cell cycle regulator A (HIRA) induces megakaryocyte differentiation of leukemia cells. (A) Control and *HIRA‐sh* K562 cells were cultured continuously for 10 d. Micrograph shows the cell morphology of control and *HIRA‐*sh K562 cells at day 7. On the 7th day post‐puromycin selection, around 10.33% (±0.98%) of enlarged cells were visible in *HIRA*‐sh K562 cells (bottom left panel, red arrows). Similar cellular pattern was absent in control cells (upper left panel). (B) Giemsa staining for the control (upper panel) and *HIRA*‐sh K562 cells demonstrated megakaryocyte like morphology of *HIRA‐*sh cells (bottom panel). Red arrows indicate enlarged multiple lobulated megakaryocytes while white arrows indicate platelets (bottom panel). 100× magnification of control (upper panel) and *HIRA*‐sh K562 cells (bottom panel) demonstrate the multi‐lobulated cells in the latter. (C) Giemsa staining of platelets formed in K562 cells upon downregulation of HIRA, in HIRA‐sh cells. The red circle and red arrows indicate the platelets. (D) PI staining of the same set of cells. (E, F) CD41, surface marker for megakaryocytes, population was analyzed by fluorescence‐activated cell sorting (FACS). Representative image of FACS analysis for the presence of CD41 population in control and *HIRA*‐sh K562 cells. G. The graph represents the percentage of CD41^+^ population in control and *HIRA‐sh* cells. Error bar = SEM for three independent experiments. Statistical analyses were performed using Student *t* test function, **P < .01. (H) mRNA expression of surface markers for megakaryocyte and erythrocytes were determined by qRT‐PCR. Error bar = SEM for three independent experiments. Statistical analyses were performed using Student *t* test function, *P < .05, **P < .01

Upon knockdown, post‐puromycin selection on day 5 and 6, around 5% of cells appeared to be larger in size in *HIRA*‐sh population (Figure S2C, bottom panels) which increased to 10.33% (±0.98%) by day 7 (Figure 2A, red arrow, bottom panel). Similar cells types were absent in control cells (Figure S2C, upper panels; 2A upper panel). Giemsa staining proved the presence of multiple lobulated cells indicating the differentiation of *HIRA*‐sh K562 cells toward megakaryocyte lineage (Figure 2B, bottom panel, red arrows). Control cells failed to demonstrate a similar morphology (Figure 2B, upper panel). Small purple colored stained cells indicate platelets produced by *HIRA‐*sh cells, absent in control cells (Figure 2B, bottom panel, white arrows, 2C, red circle, red arrows). PI staining exhibited the presence of multiple nuclei confirming polyploidy in *HIRA‐*sh K562 cells (Figure 2D, right panel). Flow cytometry analysis demonstrated the enrichment of 4N, 8N, and 16N population in HIRA‐sh cells in comparison to control cells (Figure S2D). Next, we determined the expression of megakaryocyte‐specific cell surface markers in K562 cells. CD41 or GpIIB or Integrin alpha‐IIb is associated with megakaryocyte differentiation.^15^ FACS analysis showed a significant enrichment in CD41 population among *HIRA‐*sh K562 cells to the control cells (Figure 2E‐G). Expression of *GPIIB*/*GPIIIA* (CD41/CD61) mRNA was significantly induced in *HIRA‐*sh cells whereas the erythroid marker *GYPA* was significantly downregulated (Figure 2H). Upon ectopic expression of *HIRA* in control and *HIRA*‐sh K562 cells*,* (Figure S3A‐C) a significant reduction in *GPIIIA* and *GPIIB* expression while an upregulation in *GYPA* expression was observed in *HIRA*‐sh K562 cells (Figure S3D). So, cell morphology and cell‐surface marker analysis proved the induction in megakaryocyte differentiation upon HIRA downregulation in K562 cells. But, does this hold true for in vivo as well?

### 3.3 Mouse Hira‐knockout hematopoietic progenitors undergo megakaryocyte differentiation in vivo

**Figure 3:**
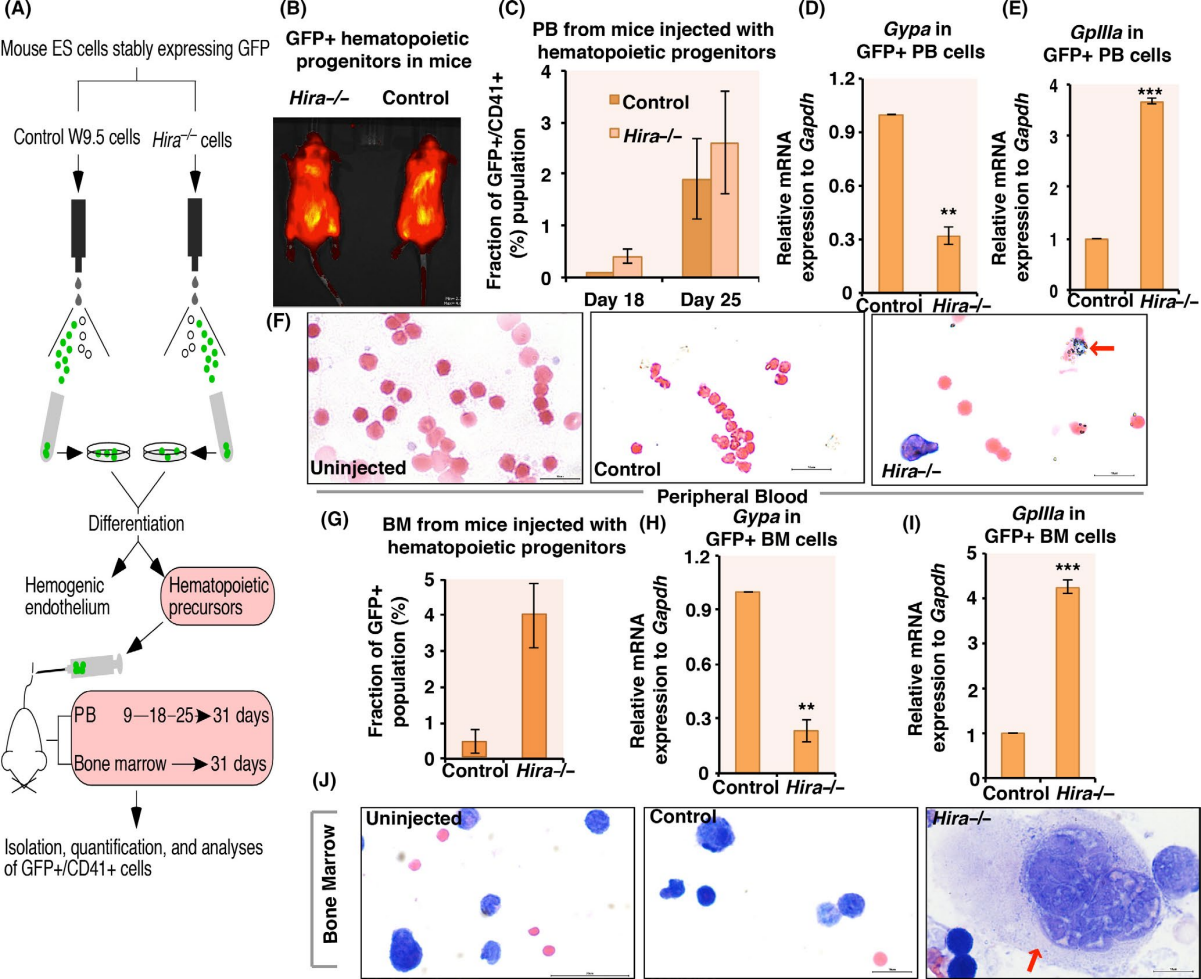
*Hira* knockout hematopoietic progenitors undergo megakaryocyte differentiation in vivo. (A) Schematic representation of tail vein injection of GFP+mouse W9.5 and *Hira* ^−/−^ hematopoietic progenitors into NOD SCID mice (N = 3) for each set of cells analyzed and the follow‐up analyses. (B) GFP‐tagged control and *Hira* ^−/−^ embryonic stem (ES) cells were differentiated to hematopoietic progenitors and injected into NOD SCID mice (N = 3) by tail vein and blood samples were extracted for analysis. (C) Peripheral blood (PB) from the mice were analyzed and sorted by fluorescence‐activated cell sorting (FACS) for the presence of GFP^+^ cells at different time points (N = 3). GFP^+^ cells were further analyzed for the expression of CD41 by FACS. The graph represents the double positive GFP/CD41 population in the PB of mice injected with control and *Hira* ^−/−^ hematopoietic progenitors. (D, E), Megakaryocyte and erythroid specific surface marker expression in GFP^+^ cells were analyzed by qRT‐PCR (N = 3). Error bar = SEM for three independent experiments. Statistical analyses were performed using Student *t* test function, **P < .01, ***P < .001. (F) Giemsa staining of PB sample isolated from mice. Red arrow indicates platelet in *Hira* ^−/−^ sample (right panel), absent in uninjected (left panel) and vector control (middle panel) cells. (G) Post‐one month injection of hematopoietic progenitors, the mice were euthanized and bone marrow from femur was isolated and analyzed for the presence of GFP^+^ population. The graph represents the GFP^+^ population present in the bone marrow of mice injected with control and *Hira* ^−/−^ hematopoietic progenitors (N = 3). (H, I) Megakaryocyte and erythroid specific surface marker expression in GFP^+^ bone marrow cells were analyzed by qRT‐PCR. Error bar = SEM for three independent experiments. Statistical analyses were performed using Student *t* test function, **P < .01, ***P < .001. J. Giemsa staining of bone marrow sample isolated from same set of mice mentioned in (F). Red arrow indicates megakaryocyte

Control (W9.5) and *Hira* ^−/−^ ES cells (a gift from Prof. Peter J Scambler) were labeled with GFP (Figure S4A) and differentiated to hemogenic endothelium followed by the generation of hematopoietic progenitors and injected into NOD SCID male mouse by tail vein (N = 3 for each set of cells) (Figure 3A and B). Zhong et al,^10^ demonstrated that tail vain injection of hematopoietic progenitors show a maximum homing capacity of these cells within a month after which it reaches a plateau. PB collected at different time points, demonstrated enriched GFP^+^/CD41^+^ population in *Hira* ^−/−^hematopoietic progenitors injected mouse (Figure 3C). *Gypa* mRNA expression was significantly downregulated whereas *GpIIIa* was significantly induced in *Hira* ^−/−^ GFP^+^ PB cells than in control cells (Figure 3D,E). Giemsa staining demonstrated the presence of platelets in PB of *Hira* ^−/−^ injected cells (Figure 3F, right panel, red arrow). Post‐one month injection of hematopoietic progenitors, BM was isolated from femur of these mice. Percentage of GFP^+^ cells was 0.65 ± 0.35% in control to 3.95 ± 0.959% in *Hira* ^−/−^ cells (Figure S4B,C,G), and the expression of *Gypa* was significantly downregulated whereas megakaryocyte specific marker *GpIIIa* was significantly induced in the BM cells isolated from *Hira* ^−/−^ cells injected mouse (Figure 3H,I). Giemsa staining confirmed the presence of induced number of megakaryocytes in BM sample of *Hira* ^−/−^ cells injected mice (Figure 3J, right panel). The percentage of megakaryocytes present in the BM of mice injected with *Hira* ^−/−^ hematopoietic progenitors was significantly more in number (Figure 3J, right panel, S4D). However, we failed to observe a difference in size of megakaryocytes from control and *Hira* ^−/−^ cells injected BM samples (Figure S4E, Figure 3J, right panel). So, HIRA downregulation could indeed induce megakaryocyte differentiation in vivo as well.

But, which factors are responsible for this differentiation?

### 3.4 Downregulation of HIRA induce MKL1 and GATA2 expression

**Figure 4:**
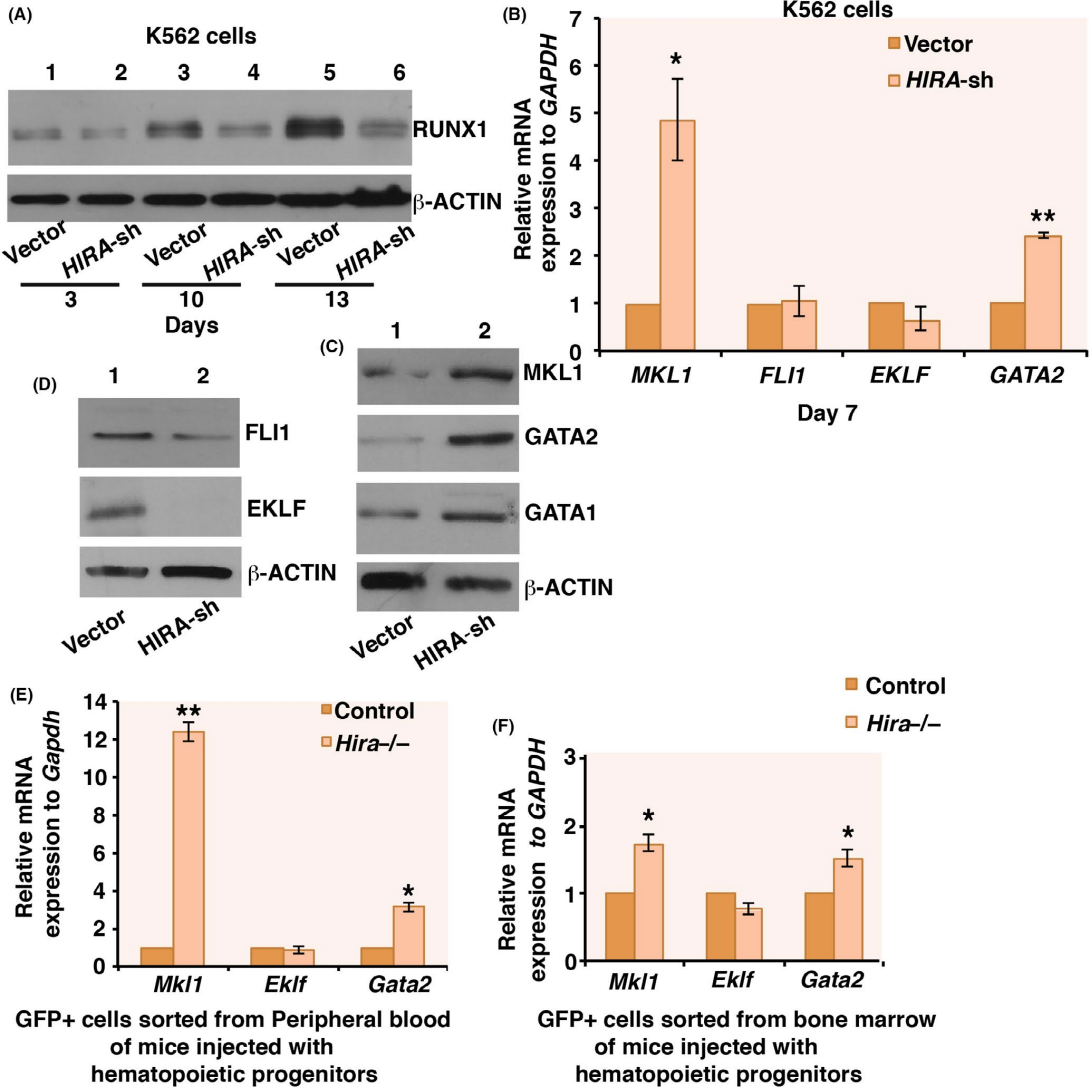
Downregulation of histone cell cycle regulator A (HIRA) induce MKL1 and GATA2 expression during megakaryocyte differentiation of chronic myeloid leukemia cells. (A) Time course analysis for the expression of RUNX1 in control and *HIRA‐sh* cells by western blot. Experiment was repeated three times and representative blot has been presented. (B). Megakaryocyte and erythrocyte specific gene expression pattern in control and *HIRA*‐sh cells were analyzed by qRT‐PCR at day 7, post‐puromycin selection. Error bar = SEM for three independent experiments. Statistical analyses were performed using Student *t* test function, *P < .05, **P < .01. (C, D) Western blot analysis for the expression of different transcription factors involved in megakaryocyte and erythroid differentiation. Experiment was repeated three times and representative blots have been presented. (E, F) Gene expression pattern of the GFP^+^ cells isolated from the peripheral blood and bone marrow of mice injected with control and *Hira* ^−/−^ hematopoietic progenitors were analyzed by qRT‐PCR. Error bar = SEM for two independent experiments. Statistical analyses were performed using Student *t* test function, *P < .05, **P < .01

Intricate network of transcription factors including RUNX1, GATA2, Friend leukemia integration 1 (FLI1), Megakaryoblastic leukemia protein‐1 (MKL1) regulates megakaryocyte differentiation.^16^ HIRA knockdown significantly reduced RUNX1 expression in *HIRA*‐sh K562 cells (Figure 4A, Figure S5A) whereas MKL1 and GATA2 was significantly upregulated in *HIRA‐*sh K562 cells (Figure 4B,C). But, no significant difference was observed in GATA1 and FLI1 expression (Figure 4B‐D). Erythroid Krüppel‐like factor (EKLF), erythroid marker, was downregulated at the protein level (Figure 4B,D).

Ectopic expression of HIRA in *HIRA*‐sh K562 cells resulted in significant downregulation of *GATA2* and *MKL1* expression (Figure S5B). Interestingly, in the GFP^+^ PB and BM cells isolated from mice injected with hematopoietic progenitors (Figure 3), significant increase in *Mkl1* and *Gata2* was observed in the samples isolated from mouse injected with *Hira* ^−/−^ hematopoietic progenitors (Figure 4E,F).

Additionally, we compared the expression of these megakaryocyte and erythroid specific genes in K562 with HL60, which express low level of HIRA (Figure 1B). It is to be noted here that we did not considered HCT116 for the same as it is a colon cancer cell line, so the context would be completely different. Also, HIRA is mutated in HCT116 cell line (Figure S5C) A significant increase in *MKL1* and *GATA2* level was observed in HL60 cells when compared to control K562 cells (Figure S5D) whereas EKLF and GYPA were significantly downregulated (Figure S5D). Upon overexpression of *HIRA* in HL60, *GYPA,* and *EKLF* expression was enhanced while *MKL1* and *GATA2* were significantly downregulated. (Figure S5E) This further proves the fact the reduced expression of HIRA could indeed set up a megakaryocyte specific machinery.

But, how HIRA knockdown could induce the expression of these markers?

### 3.5 Differential incorporation of H3.3 drives megakaryocyte differentiation

**Figure 5:**
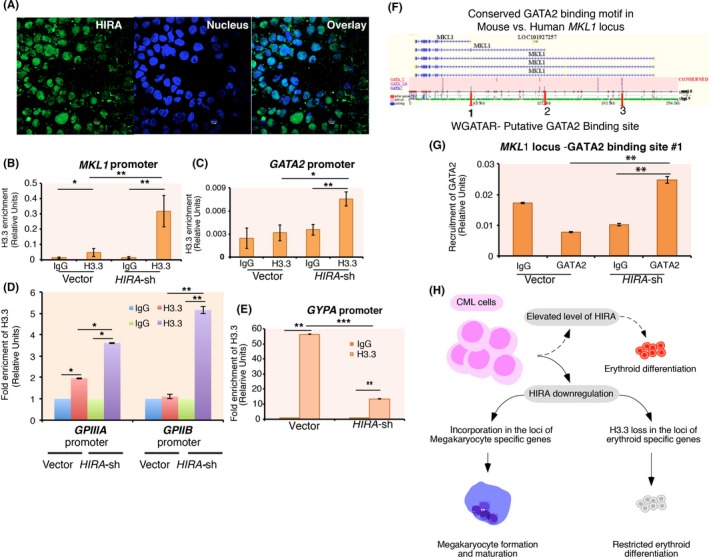
Differential H3.3 incorporation and GATA2 recruitment at *MKL1* locus contribute to the megakaryocyte differentiation. (A) Immunofluorescence analysis for the localization of histone cell cycle regulator A (HIRA) in K562 cells. (B‐E) Control and *HIRA*‐sh K562 cells were transfected with Flag/Flag‐tagged Histone H3 variant H3.3 followed by ChIP analysis. H3.3 enrichment was analyzed at the *MKL1* and *GATA2* promoter and also at the cell surface markers for megakaryocytes and erythrocytes. IgG was used as the negative control. (B, C) represent the relative enrichment whereas (D, E) represent the fold enrichment of H3.3 at the respective promoters in *HIRA*‐sh cells normalized to the empty vector expressed in K562 cells. Error bar = SEM for three independent experiments. Statistical analyses were performed using Student *t* test function, *P < .05, **P < .01, ***P < .001. (F) In silico analysis demonstrated three putative GATA2 binding motifs (WGATAR) within the *MKL*1 locus, indicated by red‐colored arrow. (G) Vector control and *HIRA*‐sh cells were subjected to ChIP analysis for the recruitment of GATA2 at the putative sites within the *MKL1* locus. Only one site demonstrated significant recruitment of GATA2 in *HIRA*‐sh cells (refer to Figure S6C,D). Error bar = SEM for three independent experiments. Statistical analyses were performed using Student *t* test function, **P < .01. (H) Model depicting downregulation of HIRA favors megakaryocyte differentiation in chronic myeloid leukemia cells

Histone cell cycle regulator A, the histone chaperone, is localized in the nucleus as per earlier reports. We determined the localization of HIRA in K562 cells. Immunofluorescence analysis demonstrated the presence of HIRA in the nucleus of K562 cells (Figure 5A). HIRA generally function as a complex comprising of calcineurin‐binding protein 1 (CABIN1) and Ubinuclein 1/2 (UBN1/2). We asked whether the knockdown of HIRA could influence the expression of these members of the complex. Gene expression analysis showed no significant change in the expression of *CABIN1,* *UBN1,* and *UBN2* in *HIRA*‐sh K562 cells compared to control cells (Figure S6A). So, next we investigated on what could be the molecular mechanism for the regulation of megakaryocyte differentiation by HIRA.

Histone cell cycle regulator A could recruit histone variant H3.3 at the chromatin resulting in either activation or repression.^17^ We observed that ectopically expressed H3.3 incorporation was significantly enriched both at the *MKL1* and *GATA2* promoter in *HIRA*‐sh cells (Figure 5B,C). However, significant loss in H3.3 level was observed at the *EKLF* promoter in *HIRA*‐sh cells compared to the control cells (Figure S6B,C). *GPIIIA,* *GPIIB* promoters were significantly enriched with H3.3 (Figure 5D) whereas significant loss in H3.3 level was observed at the *GYPA* promoter in *HIRA*‐sh cells when compared to the control cells (Figure 5E). This differential incorporation of H3.3 could account for the gene expression pattern in *HIRA*‐sh cells during megakaryocyte differentiation.

But, does MKL1 and GATA2 act independently or coordinate to induce megakaryocyte differentiation?

### 3.6 GATA2 bind to MKL1 locus upon HIRA knockdown

Comparative genomic analysis of the Evolutionary conserved region (ECR) between mouse and human, revealed the presence of three putative GATA2 binding motifs (WGATAR) within the *MKL1* locus (Figure 5F). Chromatin immunoprecipitation analysis demonstrated the recruitment of GATA2 only at #1 site within *MKL1* locus in *HIRA*‐sh K562 cells. (Figure 5G, Figure S6D,E). Thus, upon downregulation of HIRA, induced GATA2 level might facilitate its binding to the *MKL1* locus.

So, here we showed that HIRA downregulation restricts CML cell proliferation and favor megakaryocyte differentiation due to an enrichment of histone variant H3.3 at the *MKL1* and *GATA2* promoter (Figure 5H). We anticipate that HIRA could be exploited as a target for the differentiation therapy in leukemia.

## 4 DISCUSSION

Earlier we demonstrated that HIRA could regulate RUNX1, the transcription factor indispensible for the generation of hemogenic endothelium but not required in the later stages of hematopoiesis. The common myeloid progenitors, targets of RUNX1, were downregulated in the absence of HIRA.^2^ When we tried to confirm this on colony forming cell assay, we failed to observe different kinds of hematopoietic colonies, however, one particular colony type was visible (data not shown). This is to be understood here that loss in HIRA expression did not completely abolished the expression of RUNX1 as mentioned in our earlier study in hemogenic to hematopoietic transition due to the retention in a basal level of H3.3 at the *RUNX1* intronic enhancer element, implicated in this transition.^2^ So, we reasoned that the entire myeloid differentiation might not be inhibited due to the loss in HIRA expression. As a preliminary experiment, we observed that although RUNX1 was downregulated in response to *HIRA* knockdown in Kasumi 1 AML cells, GATA1 was upregulated.^2^ So, we asked would this mean that distinct progenitors could be manipulated without affecting the others. Actually, in leukemic condition, there is a repression in differentiation of different types of progenitors. So, we thought that our understanding of normal hematopoiesis as a function of HIRA could also form the basis in understanding the abnormal hematopoiesis prevalent in leukemia. From the results mentioned here, it is clear that loss in HIRA expression could impair proliferation of CML cells and could induce differentiation towards megakaryocyte fate. Although, it is interesting to see that factors like RUNX1 and FLI1 level, involved in the regulation of megakaryocyte differentiation, is repressed upon HIRA knockdown. Earlier reports suggested that overexpression of GATA2 led to megakaryocyte differentiation.^18^ Here, also we provided evidence that upon HIRA downregulation, GATA2 expression is significantly induced. But, whether the incorporation of H3.3 within the *GATA2* and *MKL1* promoter is preceded by the H3.3 recruitment at the surface marker is yet to be investigated.

But, how could loss in HIRA expression facilitate the incorporation of H3.3? Reportedly, phosphorylation of HIRA at S650 and S697 inhibits the recruitment of H3.3 at the differentiation specific loci in myoblasts and results in heterochromatin formation during senescence, respectively.^17^, ^19^ Glycogen Synthase Kinase 3 (GSK) 3 and AKT1 are responsible for the phosphorylation of the corresponding amino acids of HIRA.^19^, ^20^ Our preliminary experiment showed that HIRA downregulation resulted in loss of AKT1 expression and significant reduction in pan‐serine phosphorylation of HIRA (data not shown). So, we predict that loss in HIRA phosphorylation might induce H3.3 incorporation at the differentiation‐specific gene promoters.

Role of MKL1 in megakaryocyte differentiation has been established in CML cells. However, MKL1 also regulates smooth muscle cell differentiation.^21^ This can be correlated to the HIRA phosphorylation switch active during the differentiation of myoblasts to myotubes, a similar niche for the functioning of MKL1.^19^ The transcriptional activity of MKL1 depends on the subcellular localization, which is cell type specific. Transcription factors, could have context specific functions. In this study, we observed a significantly enhanced level of MKL1 in the myeloid cell line HL60. However, HL60 cannot undergo megakaryocyte differentiation. Basically, MKL1 associate with the filamentous actin (F‐actin) content in lymphoid and myeloid lineage immune cells and its reduced level result in widespread cytoskeletal dysfunction.^22^ Upon overexpression of HIRA, we observed shrinkage in HL60 cell size (figure not shown). This could be attributed to the fact that as HIRA overexpression resulted in significant decrease in MKL1 expression (Figure S5E) thereby leading to impaired cytoskeleton function. Although, the relation of HIRA to MKL1 and other genes discussed in this study exhibited a similar trend in ectopically expressed *HIRA* in HL60, but the function of these factors are completely context specific and hence cannot be suggestive of a parameter of linking the factors to the same extent.

Our findings demonstrated how downregulation of HIRA could facilitate the differentiation of CML cells toward megakaryocyte while inhibiting their proliferation. So, HIRA could be targeted as a molecule for enhancing the count in megakaryocytes/platelets, in cases of chronic thrombocytopenia, without causing a total inhibition in the generation of erythrocytes. Recently, a small molecule CBL0137 has been shown to inhibit the function of histone chaperone Facilitates Chromatin Transcription (FACT) and along with cisplatin, could kill patient‐derived and murine small‐cell lung cancer cell lines synergistically.^23^ Use of histone chaperone FACT inhibitor preferentially killed glioblastoma stem cells and could prolong the survival in preclinical models.^24^ Similarly, we anticipate that HIRA could be exploited, as a target for successfully approaching the differentiation therapy in leukemia, provided the full mechanism on how it could affect the normal cells is fully understood.

## CONFLICT OF INTEREST

The authors declare that we have no competing interests.

## AUTHOR CONTRIBUTIONS

AM and ATD performed all the experiments and analyzed the data. IB performed real‐time PCR and western blot analysis. AM and PCV jointly performed the animal work. AMukherjee analyzed the CCLE data and prepared schematic figures for the manuscript. LS and GN supplied the CML/AML patient samples. DD conceptualized, designed, and wrote the manuscript with final approval from all the authors.
