# Supplementary figures and images for: Histone chaperone HIRA dictate proliferation vs differentiation of chronic myeloid leukemia cells
Source: FASEB Bioadv. 2019 Aug 14;1(9):525–37. doi: 10.1096/fba.2019-00014 (PMC6996362; doi:10.1096/fba.2019-00014)

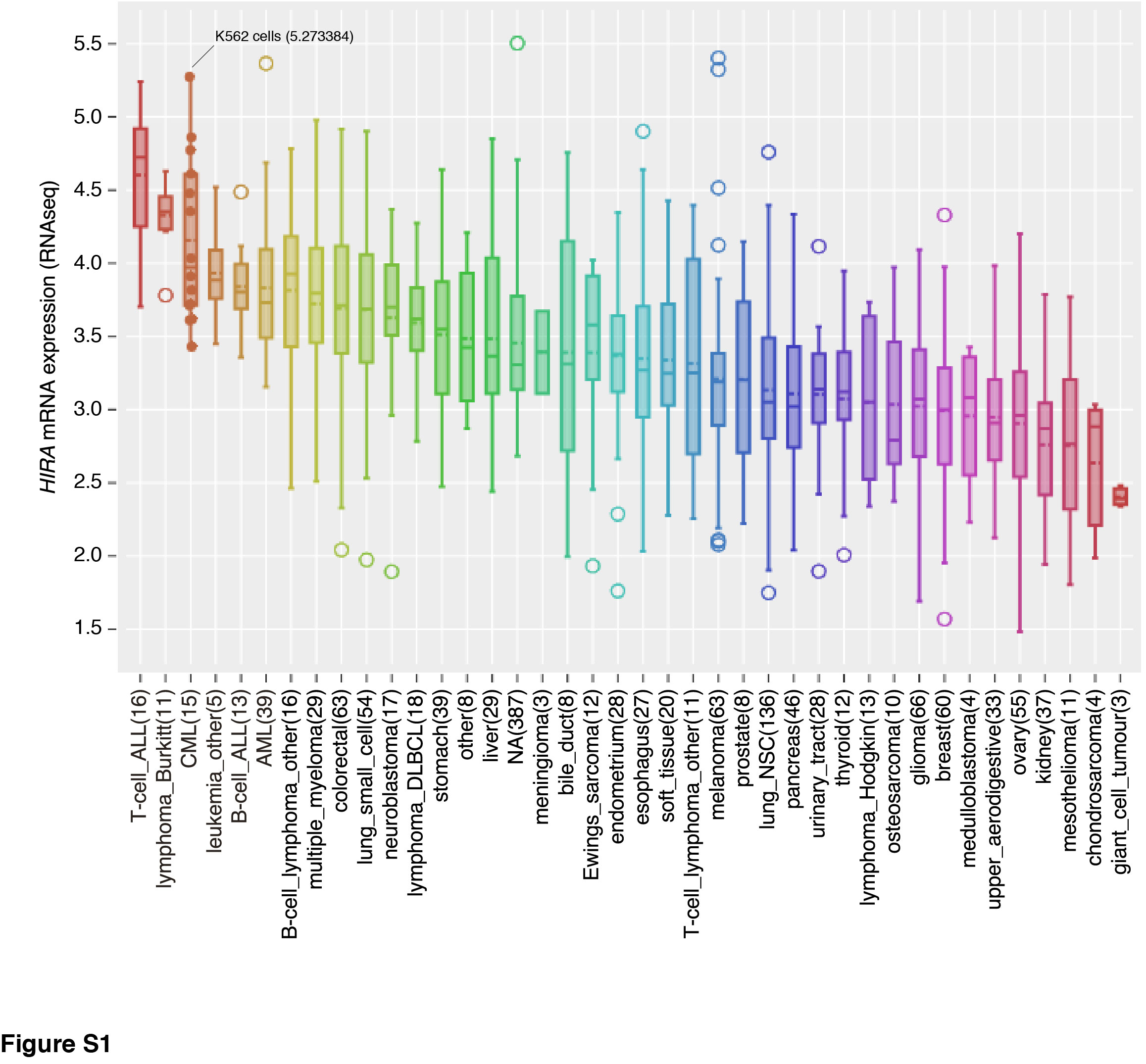

Supplement: Supplementary file 1 [file FBA2-1-525-s001.jpg]

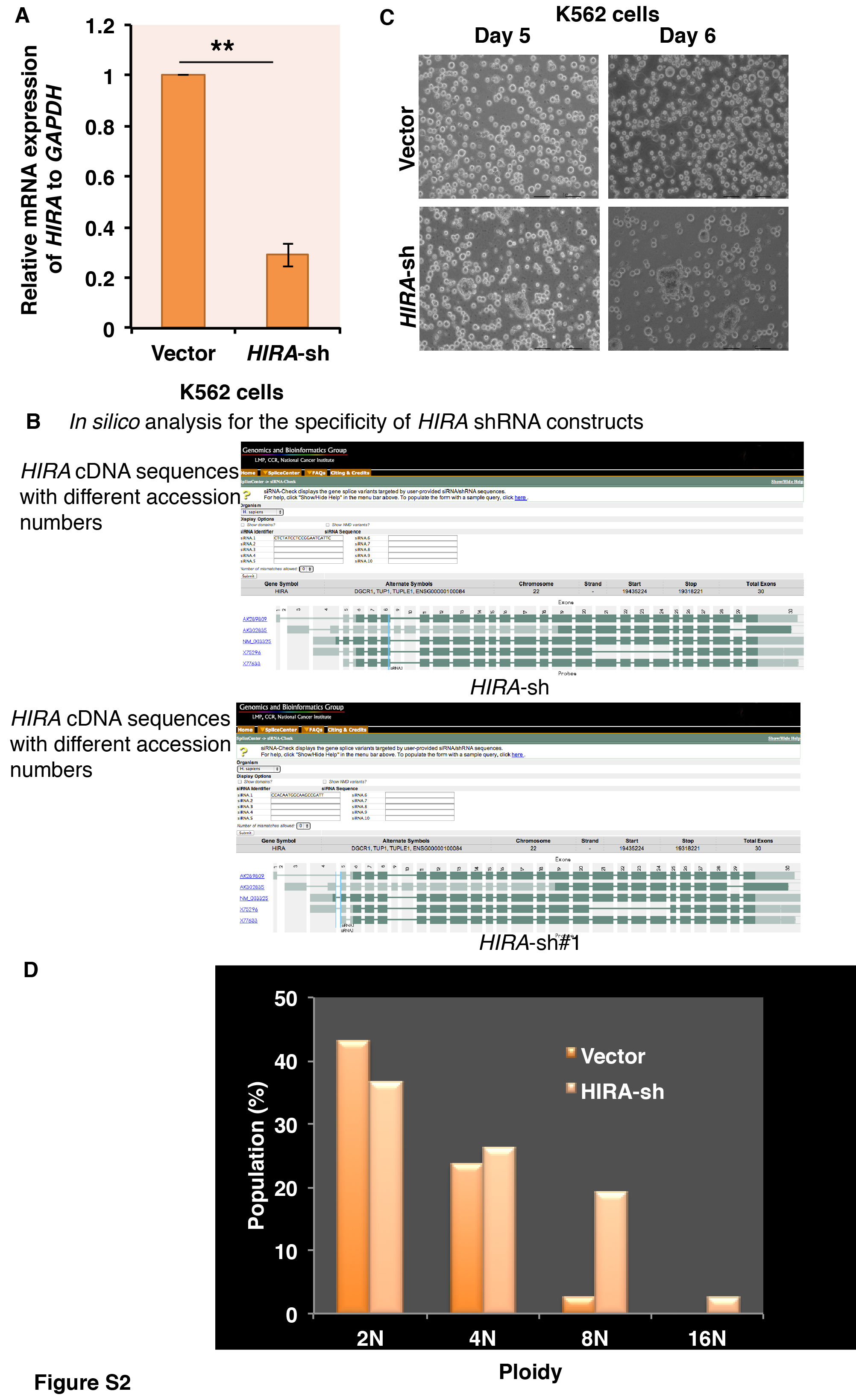

Supplement: Supplementary file 2 [file FBA2-1-525-s002.jpg]

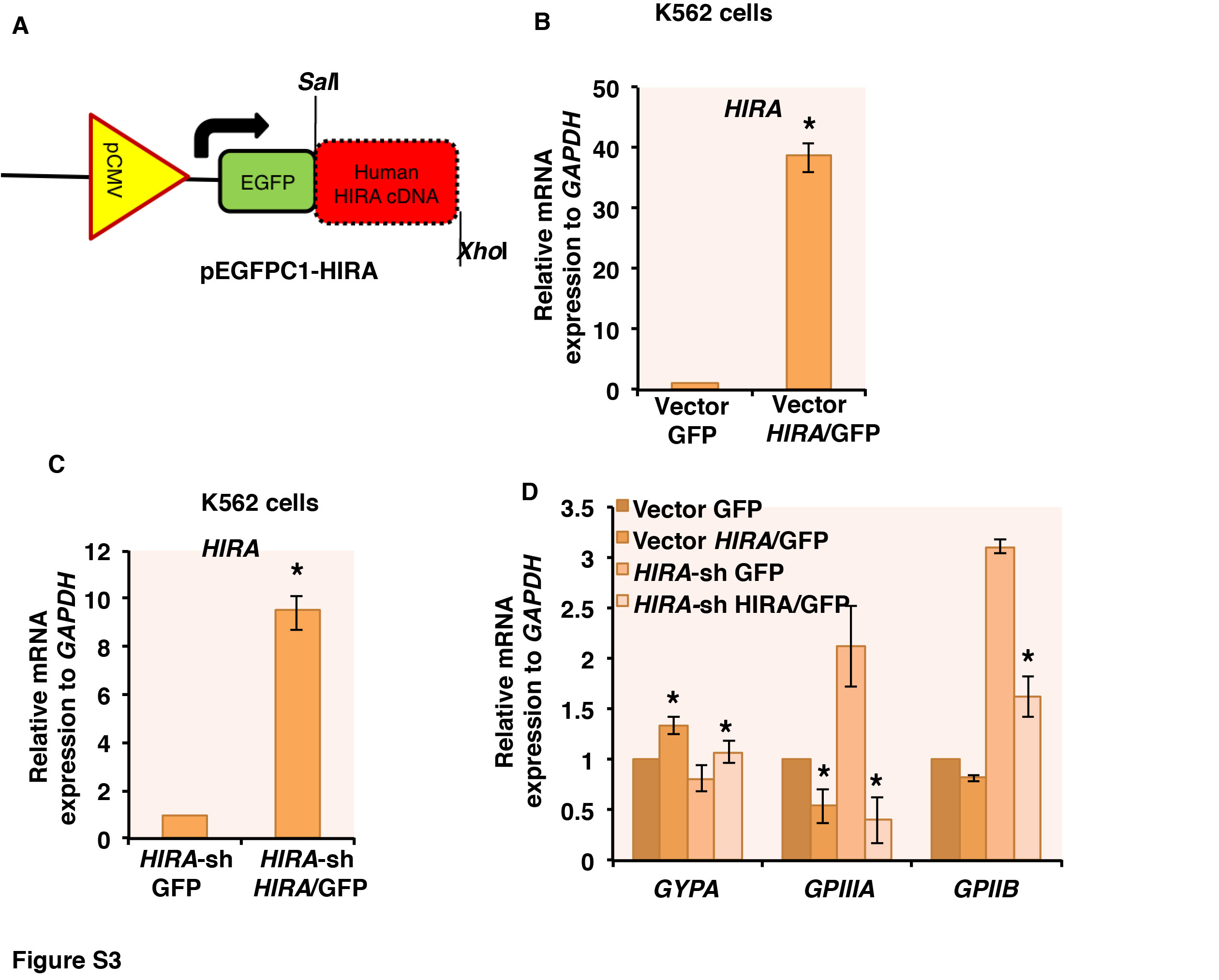

Supplement: Supplementary file 3 [file FBA2-1-525-s003.jpg]

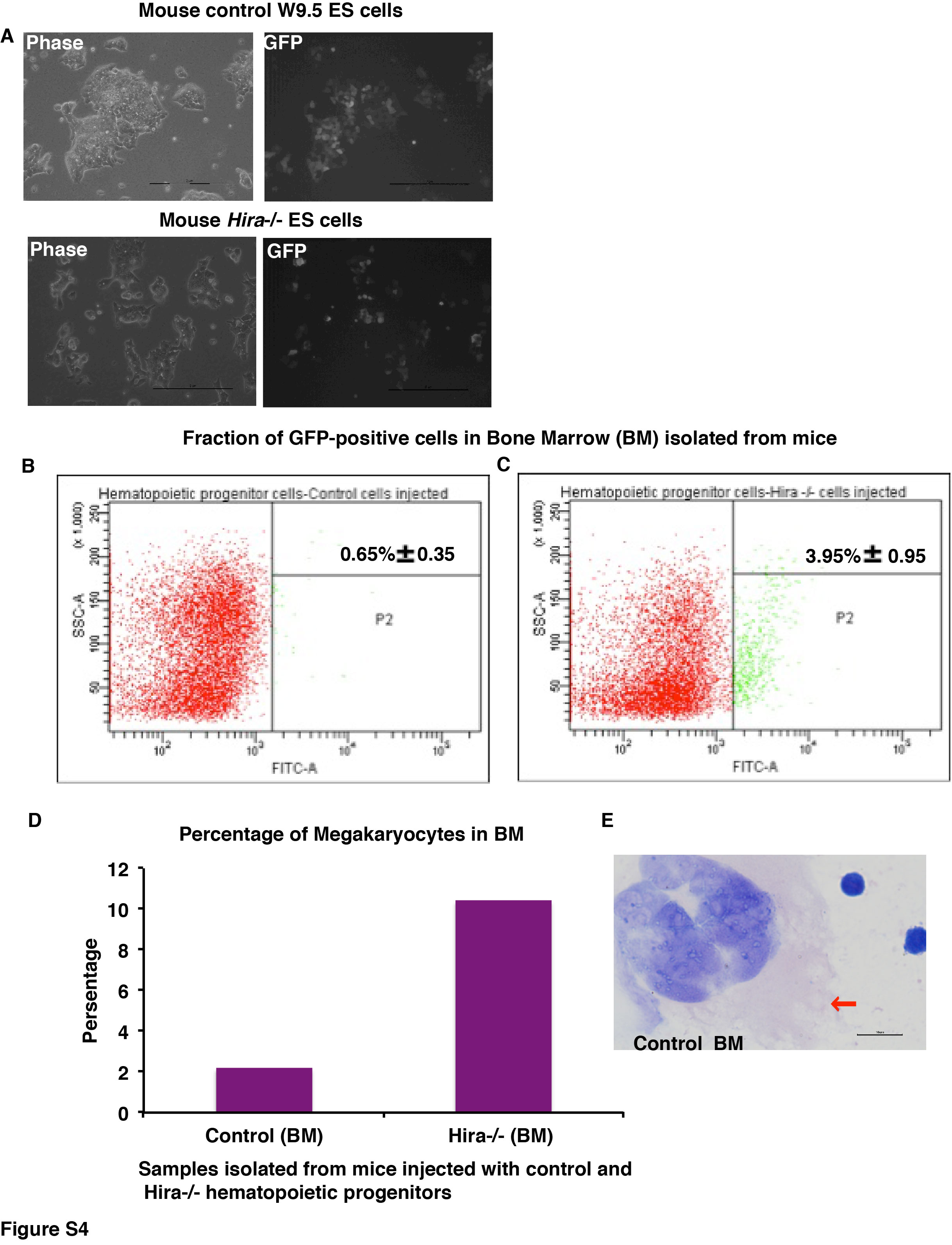

Supplement: Supplementary file 4 [file FBA2-1-525-s004.jpg]

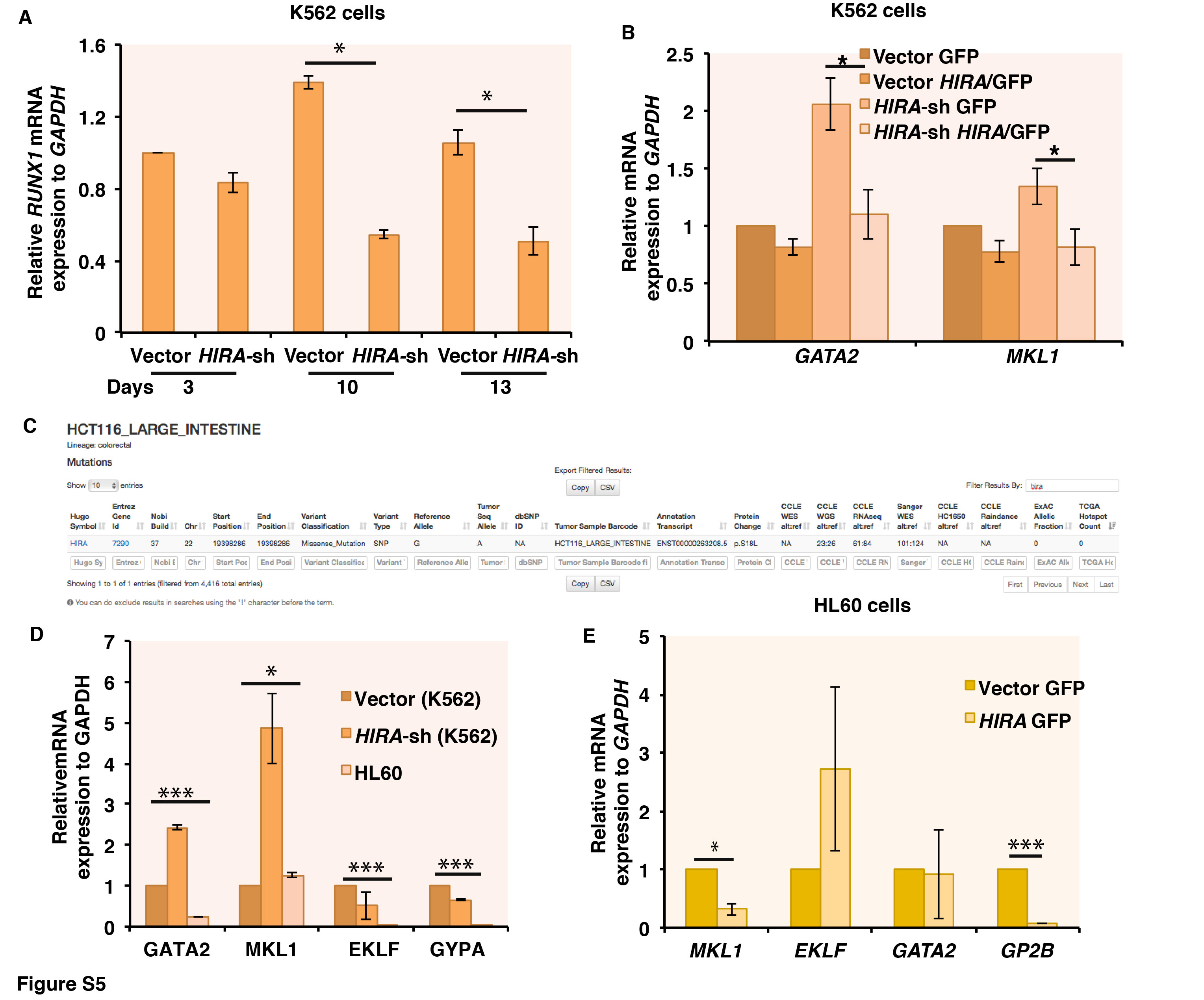

Supplement: Supplementary file 5 [file FBA2-1-525-s005.jpg]

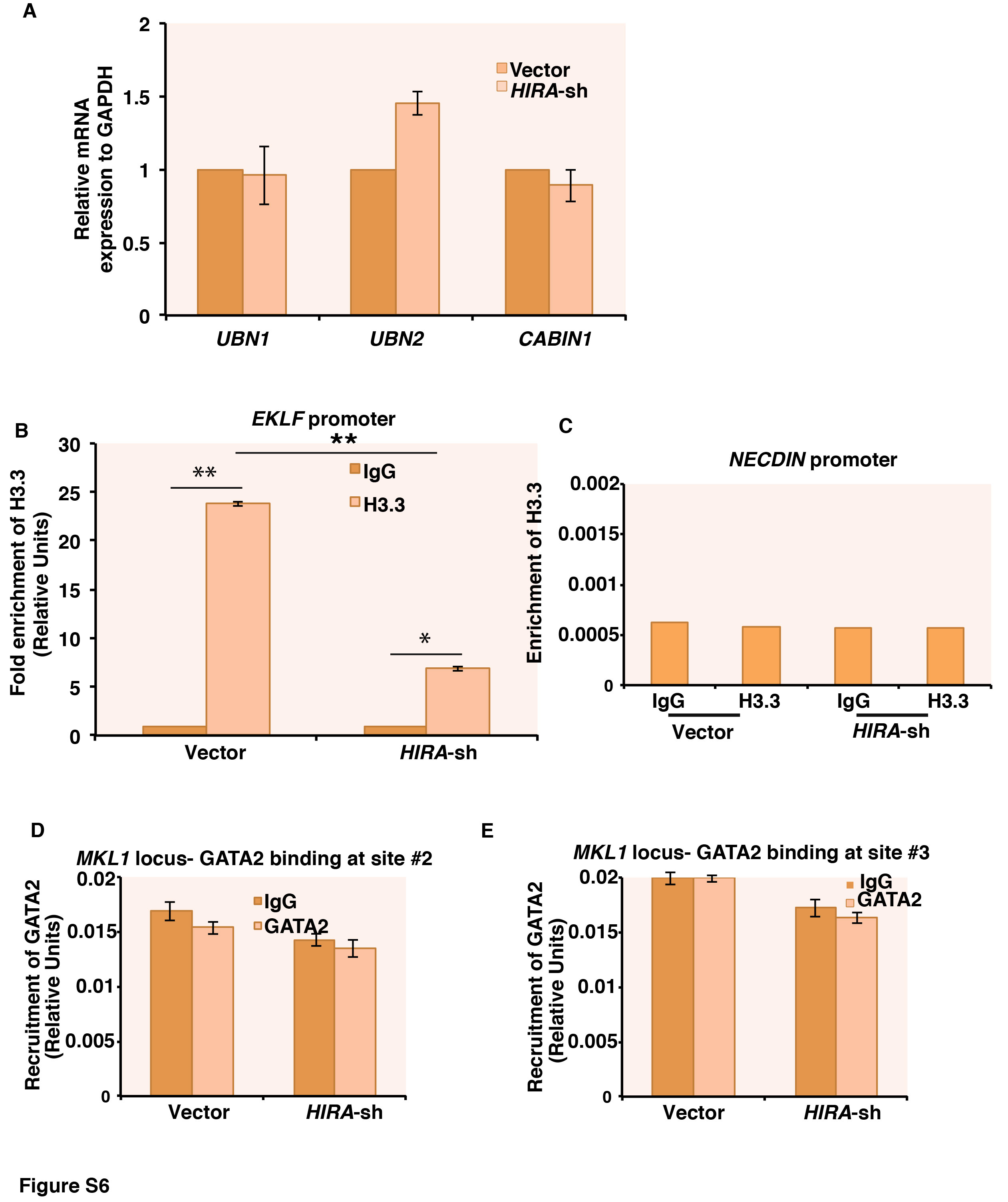

Supplement: Supplementary file 6 [file FBA2-1-525-s006.jpg]
