# Supplementary material for: Histone chaperone HIRA dictate proliferation vs differentiation of chronic myeloid leukemia cells
Source: FASEB Bioadv. 2019 Aug 14;1(9):525–37. doi: 10.1096/fba.2019-00014 (PMC6996362; doi:10.1096/fba.2019-00014)
Supplement: Supplementary file 7 [file FBA2-1-525-s007.pdf]

Histone chaperone HIRA dictate proliferation vs. differentiation of chronic myeloid leukemia cells

Supplementary Information

Supplementary Figure Legends:

Figure S1. A. The plot shows analysis of CCLE RNA seq data for the mRNA expression of HIRA in cancer cells of different lineages. The CML lineage in CCLE is composed of 15 cell lines originated from the hematopoietic and lymphoid tissue with the median of 3.972 and the maximum value of 5.273 in K562 cells. The circles represent cell lines that are outliers to the corresponding lineages. The analysis was performed using the CCLE algorithm (https://portals.broadinstitute.org/ccle/page?gene=HIRA).

Figure S2. A. qRT-PCR analysis for the expression of *HIRA* during knockdown in K562 cells. Error bar=S.E.M for three independent experiments. Statistical analyses were performed using Student t-Test function, $^{**}p<0.01$. B. Screenshot showing Splice Center, web-based online bioinformatics tool, analysis for the detection of off-targets of *HIRA* shRNA. C. Micrograph of control and *HIRA-sh* K562 cells at different days. Day 5 and day 6 (post-selection with puromycin) indicates the presence of few enlarged *HIRA-sh* cells. D. Polyploidy in K562 cells- The bar graph represents the induced polyploidy in *HIRA*-sh cell in comparison to vector K562 cells. The ploidy was measured by flow cytommetry analysis using PI.

Figure S3. A. Construct used for cloning human *HIRA* cDNA. Human *HIRA* full length cDNA (Gene ID: 7290) was amplified by PCR. The amplified *HIRA* cDNA was cloned into *Sal*I and *Xho*I sites in pEGFPC1 vector (Clonetech). B, C, D. Ectopic expression of HIRA in control and *HIRA-sh* cells to demonstrate the role of HIRA in inducing megakaryocyte differentiation. Control and *HIRA-sh* K562 cells were transfected with Empty vector or GFP-tagged HIRA and analyzed for the expression of different cell surface markers associated with differentiation. Error bar=S.E.M for three independent experiments. Statistical analyses were performed using Student t-Test function, $^{*}p<0.05$.

Figure S4. A. Micrograph showing the expression of GFP in control (W9.5) (upper panel) and $Hira^{-/-}$ (lower panel) ES cells upon lipofectamine mediated transfection of pEGFPC1 in ES cells followed by selection with G418. B, C. GFP+ control and $Hira^{-/-}$ ES cells were differentiated to hematopoietic progenitors and injected into SCID mice (N=3). At day 31, post injection, mice were sacked and bone marrow was isolated from femur bone and subjected to FACS for the detection of GFP+ cells in the bone marrow fraction. FACS analysis for the determination of GFP^{+} cells in the bone marrow isolated from mice injected with control and $Hira^{-/-}$ mouse hematopoietic progenitors respectively. D. Bar graph represents the number of megakaryocytes present in per 100 of bone marrow cells from mice injected with control and $Hira^{-/-}$ hematopoietic progenitors, observed under the microscope after Giemsa staining. E. A representative picture of megakaryocyte observed in control set of cells. Red arrow indicates megakaryocyte.

Figure S5. A. Time course analysis for the expression of *RUNX1* in control and *HIRA-sh* K562 cells determined by qRT-PCR. B. Ectopic expression of HIRA in control and *HIRA-sh* K562 cells were analyzed for the expression of different transcription factor associated with megakaryocyte differentiation. C. Screen

shot from CCLE website showing the presence of mutation in HIRA in HCT116 cells (https://portals.broadinstitute.org/ccle/page?cell_line=HCT116_LARGE_INTESTINE). D. qRT-PCR analysis for the expression of genes in control, HIRA-sh and HL60 cells. E. Ectopic expression of HIRA (using the same construct mentioned in Fig. S3A) in HL60 followed by gene expression analysis by qRT-PCR. Error bar=S.E.M for three independent experiments. Statistical analyses were performed using Student t-Test function, *p<0.05, ***p<0.001.

Figure S6. A. Control and *HIRA-sh* K562 cells were analysed for the expression of HIRA-complex factors by qRT-PCR. B. Control and *HIRA-sh* K562 cells were transfected with Flag/Flag-tagged H3.3 followed by ChIP assay for the determination of H3.3 level within the *EKLF* promoter. C. *NECDIN* promoter was used as the negative control for H3.3 incorporation. D, E. Quantitative ChIP analysis for the recruitment of GATA2 within the *MKL1* locus (site 2 and 3) in control and *HIRA-sh* K562 cells. IgG was used as the negative control. Error bar=S.E.M for three independent experiments.

Supplementary Tables:

Table S1: Primers for qRT-PCR analysis

Gene	Forward (5’ - 3’)	Reverse (5’ - 3’)
hHIRA	GGAGACCAGCATCACCAA	CATGGCATGGGCAGACAC
hGAPDH	CACCAGGGCTGCTTTTAACTCTGGTA	CCTTGACGGTGCCATGGAATTTGC
hMKL1	CAAGTCCACCCCCACACTC	CTTGCTGCGCTGTGACTTCT
hGATA2	GACCACTCATCAAGCCCAAG	TCTGACAATTTGCACAACAGG
hEKLF	CTTGCCCTCCATCAGCAC	CATGTCCTGCGCCTCTTC
hFLI1	CACACCGACCAATCCTCAC	GTTATTGCCCCAAGCTCCTC
hGPIIIa	GGACAAGCCTGTGTCACCAT	CCTGGTCAGTTAGCGTCAGC
hGPIIb	CTCGACCGGGATGGCTAC	CACTCTGACCCAGGAACACC
hGYPA	GTGATGGCTGGTGTTATTGG	CAGGTGAGGGGAGAGGTTTT
mMkl1	GAAGAATATCCTGCCTGTGGA	GAGGAACTGTCTGCTACCTTTG
mGpIIIa	ACAGAGCGTGTCCCGTAATC	GCAATATGGGTCTTGGCATC
mGata2	AAAGGGGCTGAATGTTTCG	GCGTGGGTAGGATGTGTC
mEklf	CTGAGACTGTCTTACCCTCCATC	CCAAATCCTGCGTCTCCTC
mFli1	TGTCAAGCGGGAGGGGTATGACC	TAGTTCATGGGGTTGGCTTC
mGypa	CCAAGAAGAGCATTCACCATC	CCTACAGTTGAAGCCACCACA
mGapdh	TGCCCCCATGTTTGTGATG	TGTGGTCATGAGCCCTTCC
hCABIN1	CACAAAACCCAGACAAAGGAG	GCCTTGTGGTACAATGCAAA
hUBN1	CGAAAATTTGAAGAAAAA	ACCCCATTTTATTCGATCAAGTCC
hUBN2	TGATATTACAGACAACCAAAAGCAC	CCTTCCTCTTTCCGCTTCC

Human specific primer has been indicated by h; mouse specific primers has been indicated by

Table S2: Antibodies used in the study

Primary Antibody	Company	Catalog No	Dilutions
HIRA	Millipore<br>Abcam	04-1488<br>ab20655	1:1000<br>1:200 (IF)
RUNX1	Santa-cruz	S365644	1:600
GATA1	Abcam	ab11852	1:1000
β Actin	Sigma	A5441	1:2000
GATA2	Abcam	ab8255	1:1000
EKLF	Abcam	ab2483	1:1000
CYCLIN D1	Santacruz	sc753	1:2000
PCNA	Santacruz	sc7907	1:3000
FLI1	Abcam	ab15289	1:1000
MKL1	Abcam	ab49311	1:1000
BrdU	BD Biosciences	347580	1:75

Secondary Antibody	Company	Catalog No	Dilutions
Goat Anti mouse IgG	Santa Cruz	sc-2005	1:2000
Peroxidase Affinipure Goat Anti Rabbit IgG	Jackson Immuno Research	111035144	1:7000<br>1:10,000
Peroxidase AffiniPure Donkey anti goat IgG	Jackson Immuno Research	705035003	1:7000
Goat Anti-mouse Alexa Fluor 568	Invitrogen	A-11004	1:100

FACS Antibody	Company	Catalog No	Amount
PE rat anti-mouse CD41	BD- Pharmingen	558040	0.5µg/100µl 1XPBS for 10^6 cells
FITC mouse anti human CD41	BD- Pharmingen	555469	0.5µg/100µl 1XPBS for 10^6 cells

Table S3: Quantitative ChIP primers

Gene	Forward	Reverse
hGATA2B MKL1_1	GTGAGAGGAAGTTGCGAAGG	GGTTTTGAGCAAAAGGCAAG
hGATA2B MKL1_2	AAAAGTAAAAGAAACCACTGAAAAA	CAGTCAACACAGGGGAGGA
hGATA2B MKL1_3	CATTCCAAAGTCTGAACAAGAATAA A	CCCAGAGATTGATGGTGAAA
hMKL1 Promoter	ATCCGTCATGACTCTACTGGAAC	ACTGTACTGCCATCATTAACATCT C
hGPIIIA Promoter	GGGTGAATGTGTCCCAAGAA	GTGCTTAGAAAGGCCAGGAG
hGYPA Promoter	GGCGCTTAACAACTTGCATC	TTATCTTCCAGGCCCACCTT
hGPIIB Promoter	CCTAGTCGACGTCTAGAGGCTATTG	AGTTCCCACCACCGGAAGTT
hEKLF Promoter	GCAGCCGAGGAAGAGGAG	GCGGTCAGTGTGCTGATG
hGATA2 Promoter	GTGAGCGCCAGGAAGGTAG	GCGGCAGGCAATAGACAGA
hNECDIN promoter	GGTCCTGCTCTGATCCGAAG	GGGTCGCTCAGGTCCTTACTT
